# Recurrence risk stratification based on Epstein–Barr virus DNA to identify enlarged retropharyngeal lymph nodes of nasopharyngeal carcinoma: A model-histopathologic correlation study

**DOI:** 10.3389/fmed.2022.996127

**Published:** 2022-12-01

**Authors:** Minjie Mao, Xueping Wang, Sharvesh Raj Seeruttun, Peidong Chi, Kewei Huang, Wen Liu, Wencheng Tan

**Affiliations:** ^1^Department of Laboratory Medicine, Sun Yat-sen University Cancer Center, Guangzhou, Guangdong, China; ^2^State Key Laboratory of Oncology in South China, Collaborative Innovation Center for Cancer Medicine, Guangdong Key Laboratory of Nasopharyngeal Carcinoma Diagnosis and Therapy, Sun Yat-sen University Cancer Center, Guangzhou, Guangdong, China; ^3^Department of Gastric Cancer, Sun Yat-sen University Cancer Center, Guangzhou, Guangdong, China; ^4^Department of Endoscopy, Sun Yat-sen University Cancer Center, Guangzhou, Guangdong, China

**Keywords:** nasopharyngeal carcinoma, retropharyngeal lymph nodes, recurrence, prediction model, EBV

## Abstract

**Background:**

Accurate assessment of the nature of enlarged retropharyngeal lymph nodes (RLN) of nasopharyngeal carcinoma (NPC) patients after radiotherapy is related to selecting appropriate treatments and avoiding unnecessary therapy. This study aimed to develop a non-invasive and effective model for predicting the recurrence of RLN (RRLN) in NPC.

**Materials and methods:**

The data of post-radiotherapy NPC patients (*N* = 76) with abnormal enlargement of RLN who underwent endonasopharyngeal ultrasound-guided fine-needle aspirations (EPUS-FNA) were examined. They were randomly divided into a discovery (*n* = 53) and validation (*n* = 23) cohort. Univariate logistic regression was used to assess the association between variables (magnetic resonance imaging characteristics, EBV DNA) and RRLN. Multiple logistic regression was used to construct a prediction model. The accuracy of the model was assessed by discrimination and calibration, and decision curves were used to assess the clinical reliability of the model for the identification of high risk RLNs for possible recurrence.

**Results:**

Abnormal enhancement, minimum axis diameter (MAD) and EBV-DNA were identified as independent risk factors for RRLN and could stratify NPC patients into three risk groups. The probability of RRLN in the low-, medium-, and high-risk groups were 37.5, 82.4, and 100%, respectively. The AUC of the final predictive model was 0.882 (95% CI: 0.782–0.982) in the discovery cohort and 0.926 (95% CI, 0.827–1.000) in the validation cohort, demonstrating good clinical accuracy for predicting the RRLN of NPC patients. The favorable performance of the model was confirmed by the calibration plot and decision curve analysis.

**Conclusion:**

The nomogram model constructed in the study could be reliable in predicting the risk of RRLN after radiotherapy for NPC patients.

## Introduction

Nasopharyngeal carcinoma (NPC) is a malignant tumor originating from the nasopharyngeal epithelium. It is usually associated with Epstein–Barr virus (EBV) infection (1). Compared with other head and neck primary malignancy, NPC has a high propensity to metastasize to cervical lymph nodes, and enlarged neck nodes are seen in a large fraction of patients (2). NPC responds well to radiotherapy, but disease recurrence and metastasis are still the bottlenecks that hinder the cure rate of NPC (3–5). Luo et al. (6) found that neoplastic spindle cells, which generates cancer stem cells (CSCs) and Epithelial-mesenchymal transition (EMT) properties, is likely to account for the predominant clinical characteristics of this disease. Several reports have showed the EBV oncoprotein LMP1 may be the dominant cause of facilitating the histogenesis and aggressiveness of spindle cells in NPC (7–9). The retropharyngeal lymph node (RLN) is the most commonly involved first-echelon lymph node region in NPC due to the extensive nasopharyngeal lymphatic network (10). Approximately 50% of NPC patients have enlargement of RLN after the completion of treatment (11, 12). RLN metastasis in NPC has an essential bearing on radiotherapy treatment planning (13–15). According to the National Cancer Comprehensive Network (NCCN) guidelines, surgery is recommended as the primary choice for resectable recurrent tumor lesions. RLN recurrence in NPC is very difficult to remove *via* surgery because of its location in the retropharyngeal and parapharyngeal space that is closely related to cranial nerves, the internal jugular vein, and the internal carotid artery. For recurrent RLN, radiotherapy is commonly used. However, salvage radiotherapy presents a high rate of severe complications, such as massive nasopharyngeal hemorrhage, nasopharyngeal mucosal necrosis, radiation encephalopathy, and trismus, and a >50% rate of ≥grade three radiotoxicities (16, 17). Thus, it is crucial to confirm the nature of the enlarged RLN in NPC after radiotherapy as it is related to selecting appropriate treatments and avoiding unnecessary therapy.

Magnetic resonance imaging (MRI) is critical for evaluating malignant retropharyngeal lymph nodes (18–20). Previous studies focused on the size of RLN metastasis diagnosis based on a minimum axial diameter (MAD) of 5 mm (21–23). Zhang et al. (24) and Li et al. (25) found that MAD > 6 mm was a more accurate prognosticator of RLN metastasis from NPC. However, large nodes can be reactive and non-metastatic, while small nodes can contain metastases and are difficult to resect (24). In our previous study (26), we proposed a novel minimally invasive technique termed endonasopharyngeal ultrasonography-guided fine-needle aspiration (EPUS-FNA) for sampling tissues from RLN. The aspiration smear was sent for aspiration cytology, and the tissue was sent for pathological examination. It is an effective method for diagnosing RLNs metastasis in patients with NPC, but its invasiveness and associated trauma limit its routine use. Therefore, more non-invasive and easy-to-used tool for assessing RLN recurrence are urgently needed.

Growing evidence revealed that the plasma Epstein–Barr virus DNA (EBV DNA) may be released from tumor cells during the process of apoptosis or generated from viral replication and different EBV antigens are expressed at different stages of infection (27–32). Ma et al. (31) analyzed the relationship between the plasma EBV DNA level with the delineated tumor volume and with tumor metabolic activity by PET/CT scan, and showed that plasma EBV DNA level reflects overall tumor load. Another remarkable finding is that, the levels of plasma EBV DNA in patients with NPC recurrence were higher than the levels of those who remained in continuous clinical remission (27, 33). Thus, measuring plasma EBV DNA has been shown to provide an almost real-time readout for monitoring the recurrence, prognostication, treatment response prediction and disease surveillance of NPC (34–36). However, the relationship between EBV-DNA and recurrent RLN has not been reported.

In this present study, we aimed to develop a predictive model using magnetic resonance imaging characteristics and EBV DNA for the identification of high risk RLNs for possible recurrence.

## Materials and methods

### Patients

A total of 76 NPC patients with RLN enlargement after treatment who underwent EPUS-FNA biopsy at the Sun Yat-sen University Cancer Center (Guangzhou, China) between April 2015 and December 2021 were selected. The inclusion criteria were: (1) NPC patients without distant metastasis, (2) had one or more enlarged RLNs assessed with MRI during regular follow-up >6 months after the end of radiotherapy, (3) absence of additional known head and neck cancers or acute inflammation, (4) had no chemotherapy, radiotherapy, immunotherapy, or salvage surgery between the completion of radiotherapy and the MRI diagnosis of suspicious RLN metastasis. Patients enrolled in the study were randomly divided into a discovery (*n* = 53) and the validation (*n* = 23) group. All patients provided written informed consent. The Institute Research Ethics Committee of the Sun Yat-sen University Cancer Center approved this study (NO: SL-B2022-687-01).

### Treatment

All eligible patients received radiotherapy as their primary treatment with or without chemotherapy. The target volume delineation was referred to the International Commission Radiological Units Guidelines. Gross tumor and RLN were included within the primary gross target volume at our cancer center. The prescribed dose was 66–72 Gy for the primary target and 60–66 Gy for the involved cervical lymph nodes. Five daily fractions per week were given to the patients, and the radiotherapy lasted for 6–7 weeks.

### Diagnostic criteria for recurrence of retropharyngeal lymph node

All patients underwent EPUS-FNA. If pathology or cytology indicated cancer cell-negative, the patient were recommended for a second EPUS-FNA. If the results were also negative, the patients were then closely followed up every 3 months using MRI. During follow-up, if a suspicious RLN was stable for 6 months after the latest EPUS-FNA, they were still regarded as cancer cell-negative and underwent further follow-up. The diagnostic criteria for RRLN in our study were defined as: an RLN with pathologic or cytologic confirmation, or progressive enlargement of RLN during MRI follow-up.

### Magnetic resonance imaging protocol and Epstein–Barr virus DNA measurement

All patients underwent MRI with a 1.5-T system (GE Discovery MR750; General Electric Healthcare, Waukesha, WI, USA), with a head and neck-combined coil for the nasopharyngeal scans. The same MR imaging sequences for all patients were obtained, including axial, coronal and sagittal T1-weighted and T2-weighted images before intravenous injection of the contrast material (Magnevist; Bayer Schering Pharma, Berlin, Germany). The images were assessed by two experienced radiologists. Any disagreements were resolved by mutual discussion. The section thicknesses and intersection gaps were 5 and 1 mm for the axial plane, and 6 and 1 mm for the coronal and sagittal planes. The following MR features are recorded: minimum axial diameter (MAD), lymph nodes signal, irregular margin, central nodal necrosis (CNN), and abnormal enhancement. MAD was measured at the widest diameter of the lymph node on T1 axial images without fat suppression. EBV DNA results were obtained within 1 month prior to EPUS-FNA biopsy which was detected by fluorescence quantitative PCR using a commercial kit (Targene, Anhui). Samples of peripheral blood (3 ml) were collected in an EDTA tube from all NPC patients and were centrifuged at 1,600 × g for 15 min for isolation of plasma and PBC. Plasma DNA was extracted using the QIAamp Blood Kit (Qiagen, Hilden, Germany) and a total of 500 μl of the plasma samples was used for DNA extraction per column, and a final elution volume of 50 μl was used to elute the DNA from the extraction column. A real-time quantitative PCR system was developed for plasma EBV DNA detection toward the *Bam*HI-W region of the EBV genome. The sequences consisted of the amplification primers W-44F (5′-AGT CTC TGC CTC AGG GCA-3′) and W-119R (5′-ACA GAG GGC CTG TCC ACCG-3′) and the dual-labeled fluorescent probe W-67 T [5′-(FAM) CAC TGT CTG TAA AGT CCA GCC TCC(TAMRA)-3′] (37).

### Statistical analysis

The Statistical Product and Service Solutions (SPSS, ver. 22.0; IBM, Chicago, IL, USA) and R (ver. 3.5.1^1^) software were used for statistical analyses. The application value of EBV DNA was evaluated by the area under the receiver operating characteristic (ROC) curve. Univariate and multivariate logistic regression analyses were used to analyze the risk factors associated with RRLN. A nomogram was conducted using statistically significant features identified in multivariate analysis. We tested the accuracy of the nomogram by graphical calibration using the observed outcome plotted against the predicted probability of the outcome obtained from the fitted logistic regression model in both the discovery and validation cohorts. Discrimination of the model was summarized using the area under the ROC curve. Furthermore, we plotted decision curves to assess the benefits of the nomogram-assisted decisions in a clinical context. A two tailed *P*-value < 0.05 was considered statistically significant.

## Results

### Patient characteristics

The data of a total of 76 NPC patients were retrieved and assessed. There were 53 patients in the discovery cohort, and 40 (75.4%) had detectable RRLN. In the validation cohort, 23 patients were screened, and 17 (73.90%) patients had detectable RRLN. The study population comprised of 53 males and 23 females with NPC. The median age was 46 (range: 39–53) years. Based on 8th edition of American Joint Committee on Cancer TNM classification, there were two (2.63%) patients classified as stage I, nine (11.84%) as stage II, 37 (48.68%) as stage III, and 26 (34.21%) as stage IV. There are no significant differences between the two cohorts. Their basic characteristics are given in Table 1.

**TABLE 1:** Patient characteristics in the discovery and validation cohort.

Variable	Total	Discovery cohort	Validation cohort	*P*
	(*n* = 76)	(*n* = 53)	(*n* = 23)	
Age, median (IQR), years	46 (39–53)	45 (39–53)	47 (41–52)	0.46
**Sex**				
Male	53	46	17	0.17
Female	23	7	6	
**T stage**				
T1	8	6	2	0.82
T2	12	9	3	
T3	38	26	12	
T4	16	10	6	
***N* stage**				
N0	9	4	5	0.12
N1	25	20	5	
N2	28	21	7	
N3	12	6	6	
**Clinical stage**				
I	2	1	1	0.46
II	9	8	1	
III	37	26	11	
IV	26	16	10	
**Retropharyngeal lymph node**				
Positive	57	40	17	0.89
Negative	19	13	6	
**Smoking**				
Yes	16	10	6	0.36
No	47	35	12	
**Alcohol behavior**				
Previous/Current	8	6	2	0.81
Never	55	39	16	
**Family history of cancer**				
Yes	13	8	5	0.35
No	51	38	13	
**EBV-DNA (copy/ml)**				
≤200	40	28	12	0.96
>200	36	25	11	
**EBER**				
Positive	52	37	15	0.76
Negative	8	6	2	

RRLN, recurrence retropharyngeal lymph node; MAD, minimum axis diameter.

### Model construction

**FIGURE 1:**
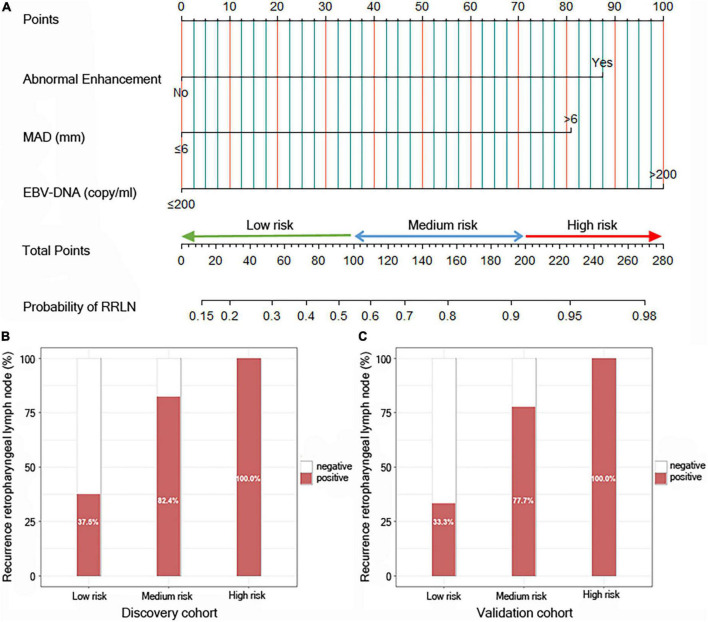
Risk calculator for recurrence of retropharyngeal lymph node (RRLN) in nasopharyngeal carcinoma (NPC) patients with post-radiotherapy. **(A)** Nomogram predicting the risk of RRLN. Patients can be divided into three groups based on their scores: low-risk group (total points: ≤100), middle-risk group (total points: 100–200) and high-risk group (total points: >200). **(B)** Bar plot showing RRLN in the three predefined subgroups of predicted outcomes from the discovery cohort, and **(C)** the validation cohort.

To build a predictive model for RRLN, we first performed logistic regression analyses using the data of patients from the discovery cohort to study the association between clinical factors (demographics, imaging features, and blood parameters) and RRLN diagnosis. Univariate analysis showed that abnormal enhancement (*P* = 0.001), MAD (*P* < 0.001), and EBV DNA (*P* = 0.008) were significantly associated with RRLN. From the variables associated with RRLN in the univariate analyses, the final predictive model was built using parameters abnormal enhancement (*P* = 0.050, HR = 6.767), MAD (*P* = 0.039, HR = 5.860), and EBV DNA (*P* = 0.038, HR = 8.915), which were determined by multivariate analysis (Table 2). Actually based on the nomogram (Figure 1A), the first horizontal line represented the point values for each variable in vertical line, then all the corresponding points are summed to obtain the total points. Finally, from the total points we could have got the probability of RRLN. A nomogram was developed and three subgroups for predicting RRLN, based on the total points, were delineated: low risk (0–100), medium risk (100–200) and high risk (>200). We tested comparing for the three predefined subgroups of predicted RRLN in the discovery and validation cohorts: low risk (37.5, 33.3%), medium risk (82.4, 77.7%), and high risk (100, 100%) in Figures 1B,C.

**TABLE 2:** Univariate and multivariate logistic regression to predict RRLN.

	Univariate analysis		Multivariate analysis	
		
Characteristics	HR (95% CI)	*P*	HR (95% CI)	*P*
**Sex**				
Male/Female	2.118(0.231−19.438)	0.499		
**T stage**				
T1-2/T3-T4	0.400(0.076−2.099)	0.268		
***N* stage**				
N0-1/N2-3	0.752(0.203−2.782)	0.669		
**Smoking**				
Yes/No	1.185(0.208−6.744)	0.848		
**Alcohol behavior**				
Yes/No	1.500(0.155−14.557)	0.725		
**Family history of cancer**				
Yes/No	0.931(0.159−5.446)	0.937		
**Abnormal enhancement**				
Yes/No	10.500(2.338−47.153)	0.001	6.767(0.997−45.933)	0.05
**Necrosis**				
Yes/No	0.968(0.218−4.287)	0.966		
**Uneven signal**				
Yes/No	0.511(0.142−1.838)	0.3		
**Irregular margin**				
Yes/No	0.923(0.239−3.564)	0.908		
**MAD (mm)**				
≤6/>6	12.750(2.952−55.067)	<0.001	5.860(1.091−31.479)	0.039
**EBV-DNA (copy/ml)**				
≤200/>200	7.441(1.455−38.049)	0.008	8.915(1.125−70.661)	0.038
**EBER**				
Positive/Negative	2.583(0.383−17.432)	0.318		

RRLN, recurrence retropharyngeal lymph node; MAD, minimum axis diameter.

### Assessment of prediction model performance

**FIGURE 2:**
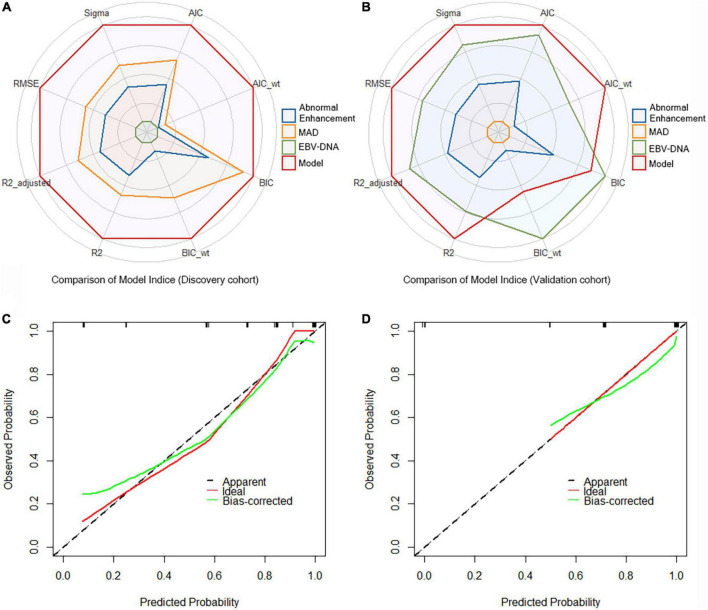
Comparison of model indices from **(A)** the discovery cohort and **(B)** the validation cohort. Calibration curves of the model for recurrence of retropharyngeal lymph node (RRLN) prediction from **(C)** the discovery cohort and **(D)** the validation cohort.

A crucial part of statistical analysis is evaluating a model’s quality and fit. We used multiple model quality parameters to evaluate the fitting of the established prediction model to the data. The six common evaluation parameters (AIC, BIC, R2, R2_adj, RMSE, and Sigma) showed that our model was the best fitting model for predicting RRLN, compared with a single indicator (Figures 2A,B). Meanwhile, the calibration plots also demonstrated good agreement between predictions and actual observations in the both discovery and validation cohorts (Figures 2C,D).

### Clinical usefulness

**FIGURE 3:**
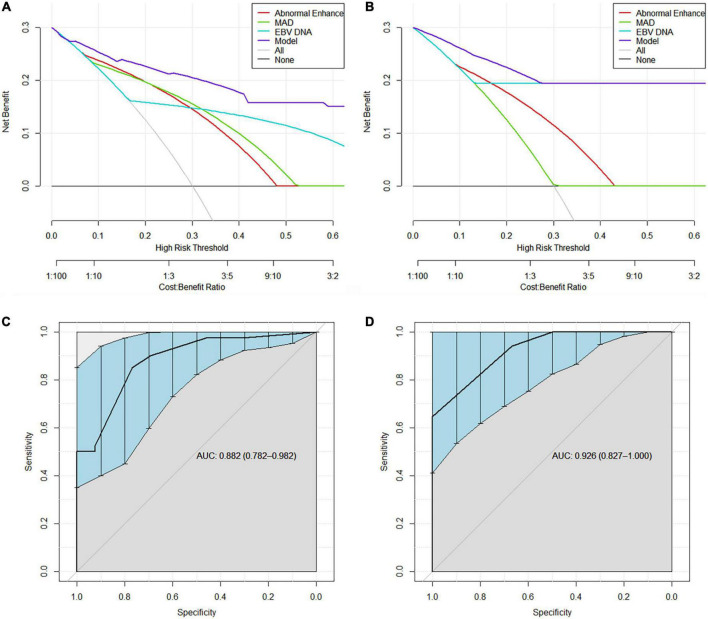
Decision curve analysis demonstrating the clinical utility in predicting recurrence of retropharyngeal lymph node (RRLN) of the model from **(A)** the discovery cohort and **(B)** the validation cohort. ROC curves demonstrating the accuracy for predicting RRLN from **(C)** the discovery cohort and **(D)** the validation cohort.

Decision curve analysis revealed that the model were applicable if the threshold probability of a patient is >10% (Figures 3A,B). The gray line represents the assumption that all patients are with RRLN, and the horizontal line represents the assumption that patients are without RRLN. DCA showed that the net benefit of the prediction model was better than a single factor (abnormal enhancement, MAD, and EBV DNA) in predicting RRLN in NPC both in the discovery and validation cohorts. Figures 3C,D illustrate that the model possesses promising accuracy for predicting RRLN. The Harrell’s concordance index (C-index) for the model to predict RRLN was 0.882 (95% CI 0.782–0.982) for the discovery cohort and 0.926 (95% CI 0.827–1.000) for the validation cohort. The detailed statistical results of the models’ performance for predicting RRLN (Table 3).

**TABLE 3:** Performance of model for predicting RRLN.

Cohort	AUC (95% CI)	SEN (%)	SPE (%)	ACC (%)	PPV (%)	NPV (%)
Discovery	0.882 (0.782–0.982)	85.0	76.9	84.9	91.9	62.5
Validation	0.926 (0.827–1.000)	88.2	66.7	82.6	88.2	66.7

RRLN, recurrence retropharyngeal lymph node; AUC, area under the receiver operating curve; CI, confidence interval; SEN, sensitivity; SPE, speci-ficity; ACC, accuracy; PPV, positive predictive value; NPV, negative predictive value; US, ultrasound; PE, physical examination.

## Discussion

To the best of our knowledge, this is the first study focusing on MRI features, EBV level to predict RRLIN in NPC patients, and further established a prediction model consisting of abnormal enhancement, minimum axis diameter (MAD), and EBV-DNA. Based on the proposed model, we divided patients into three groups: low-risk (RRLN%: 37.5%), medium-risk (RRLN%: 82.4%), and high-risk (RRLN%: 100%) groups. Compared with using MAD only to predict RRLN in the previous study, our model exhibited promising discrimination ability with a C-index of 0.882 (95% CI: 0.782–0.982) and may assist in determining the nature of abnormal enlarged RLN.

Intensity-modulated radiotherapy (IMRT) is the primary treatment modality as NPC is highly sensitive to ionizing radiation (1, 38, 39). However, around 10% of patients either have residual disease or would develop recurrence at the primary and/or regional site after IMRT treatment (40, 41). Early detection of RRLN during follow-up in patients with NPC has been shown to improve survival with salvage therapy (surgery or radiotherapy) (42, 43). Several recent internationally consensus guidelines have standardized the contouring of target and adjacent organs and dose prioritization and acceptance criteria for radiotherapy planning (44–46). However, considering the potentially severe complications of salvage treatment, assigned based on minimum axial diameter for identifying RLN metastasis has been debated. Therefore, it is necessary to identify significant features of RRLN to improve the quality of clinical decision-making.

It is currently accepted that MRI can provide superior soft-tissue contrast compared to CT and is better for differentiating between primary tumor extension and RLN metastasis than computed tomography (CT) (47). In addition, metastatic RLN lesions could shrink after the completion of RT, while the sizes of reactive nodes would usually stay stable (24). Therefore, MRI minimal axial diameter could be a reliable method for evaluating RRLN in NPC patients after treatment. Lam et al. (48) proposed a radiologic MAD of 5 mm to identify metastatic RLN. To improve the accuracy of RRLN, some investigators have suggested raising the cut-point value of MAD from 5 to 6 mm (24, 25). In our study, the most accurate size criterion of RRLNs was a MAD of 6 mm, resulting in an accuracy of 76.3%. Our results differ from the previous findings (24) suggesting that node with central necrosis was not associated with RRLN (*P* = 0.966) mainly because lymph node necrosis are seldom seen with RLN, particularly with small nodes. On the other hand, multivariate logistic regression showed that abnormal enhancement signals were independent predictors of RRLN with a diagnostic sensitivity of 85%. Structural changes of RRLN became evident on postinjection T1-weighted images, and nodal involvement on contrast enhanced MR lymphangiograms were characterized with partial or marginal enhancement of the node, indicating partial occupation by the tumor (49).

Plasma EBV DNA could also be used as a complementary surveillance method to conventional imaging for monitoring failure (1). A prospective multicenter clinical trial confirmed that post-radiotherapy plasma EBV DNA levels correlated significantly with the corresponding hazards of locoregional failure and distant metastasis (50). Our study revealed that high levels of EBV DNA (>200 copy/ml) were associated with the occurrence of RRLN and improved the diagnostic efficacy of radiology for RRLN with AUC from 0.798 to 0.882. The plasma EBV DNA is mainly derived from tumor cells, appears to correlate closely with the presence of residual tumors. Li et al. (51) proposed that ultrasound-guided cervical lymph node (CLN) FNA detection of EBV concentration may provide a new method with high sensitivity and specificity for the diagnosis of CLN metastasis. However, there are still some controversies, including the effects of sample collection, horizontal cutting, and plasma EBV. The results of our study show that no difference (*P* = 0.318) in EBER between patients with RRLN–positive and RRLN–negative disease. This result may be due to the complexity of EBV kinetics, which affects the distribution, persistence, and interchange of EBV among plasma and tissue (52). Plasma EBV DNA for predicting RRLN in NPC may be related to the ability of the virus to regulate cellular signaling pathways, block antiviral cytokines, and regulate immunosuppressive biological molecules to resist and escape host immunity (53). As a result, detection of EBV DNA provides an almost real-time readout of the tumor burden, and is useful for predicting RRLN in NPC.

The present investigation had some limitations that warrant consideration. First, the model was established based on a small sample size from a single-center and some cases were excluded because of incomplete information, which may have resulted in certain level of selection bias. Second, for patients with negative pathological results at the EPUS-FNA, they were only followed up for 6 months and were classified as stable non-recurrent lymph nodes, which may have led to mis-grouping for slow-growing tumors. Third, several novel imaging techniques such as ultrasound elastography and contrast-enhanced ultrasonography were not assessed and their adaptation may improve the prediction models in the future.

## Conclusion

This is the first study to investigate the correlation between radiologic-EBV DNA and RLN histopathologic. We also propose a predictive clinical model for the risk stratification of RRLN in NPC. This prediction tool may help identify RRLN high-risk patients who could benefit from needle biopsy or more aggressive follow-up scheme to improve their chances for longer survival.

## Data availability statement

The authenticity of this article has been validated by uploading the key raw data onto the Research Data Deposit public platform (www.researchdata.org.cn), with the approval RDD number as RDDA2022117093.

## Ethics statement

The studies involving human participants were reviewed and approved by the Institute Research Ethics Committee of the Sun Yat-sen University Cancer Center (No: SL-B2022-687-01). Written informed consent to participate in this study was provided by the participants’ legal guardian/next of kin. Written informed consent was obtained from the individual(s), and minor(s)’ legal guardian/next of kin, for the publication of any potentially identifiable images or data included in this article.

## Author contributions

WT and MM contributed to the conception and design of the study and drafted the manuscript. XW, SS, and WL contributed to the data analysis and interpretation. PC and KH participated in data collection and literature research. All authors read and approved the final manuscript.

## Footnotes

1
http://www.Rproject.org

## References

[1] Chen YP Chan A Le QT Blanchard P Sun Y Ma J. Nasopharyngeal carcinoma. *Lancet.* (2019) 394:64–80. 10.1016/S0140-6736(19)30956-0 31178151

[2] Razak AR Siu LL Liu FF Ito E O’Sullivan B Chan K. Nasopharyngeal carcinoma: the next challenges. *Eur J Cancer.* (2010) 46:1967–78. 10.1016/j.ejca.2010.04.004 20451372

[3] Tseng M Ho F Leong YH Wong LC Tham IW Cheo T Emerging radiotherapy technologies and trends in nasopharyngeal cancer. *Cancer Commun.* (2020) 40:395–405. 10.1002/cac2.12082 32745354 PMC7494066

[4] Wong K Hui EP Lo KW Lam W Johnson D Li L Nasopharyngeal carcinoma: an evolving paradigm. *Nat Rev Clin Oncol.* (2021) 18:679–95. 10.1038/s41571-021-00524-x 34194007

[5] Tang LL Chen YP Chen CB Chen MY Chen NY Chen XZ The chinese society of clinical oncology (csco) clinical guidelines for the diagnosis and treatment of nasopharyngeal carcinoma. *Cancer Commun.* (2021) 41:1195–227. 10.1002/cac2.12218 34699681 PMC8626602

[6] Luo WR Chen XY Li SY Wu AB Yao KT. Neoplastic spindle cells in nasopharyngeal carcinoma show features of epithelial-mesenchymal transition. *Histopathology.* (2012) 61:113–22. 10.1111/j.1365-2559.2012.04205.x 22486228

[7] Horikawa T Yang J Kondo S Yoshizaki T Joab I Furukawa M Twist and epithelial-mesenchymal transition are induced by the ebv oncoprotein latent membrane protein 1 and are associated with metastatic nasopharyngeal carcinoma. *Cancer Res.* (2007) 67:1970–8. 10.1158/0008-5472.CAN-06-3933 17332324

[8] Horikawa T Yoshizaki T Kondo S Furukawa M Kaizaki Y Pagano JS. Epstein-barr virus latent membrane protein 1 induces snail and epithelial-mesenchymal transition in metastatic nasopharyngeal carcinoma. *Br J Cancer.* (2011) 104:1160–7. 10.1038/bjc.2011.38 21386845 PMC3068490

[9] Luo W Yao K. Molecular characterization and clinical implications of spindle cells in nasopharyngeal carcinoma: a novel molecule-morphology model of tumor progression proposed. *PLoS One.* (2013) 8:e83135. 10.1371/journal.pone.0083135 24349446 PMC3861507

[10] Wang XS Hu CS Ying HM Zhou ZR Ding JH Feng Y. Patterns of retropharyngeal node metastasis in nasopharyngeal carcinoma. *Int J Radiat Oncol Biol Phys.* (2009) 73:194–201. 10.1016/j.ijrobp.2008.03.067 18538502

[11] Li WZ Liu GY Lin LF Lv SH Qiang MY Lv X Mri-detected residual retropharyngeal lymph node after intensity-modulated radiotherapy in nasopharyngeal carcinoma: prognostic value and a nomogram for the pretherapy prediction of it. *Radiother Oncol.* (2020) 145:101–8. 10.1016/j.radonc.2019.12.018 31931288

[12] Meng K Tey J Ho F Asim H Cheo T. Utility of magnetic resonance imaging in determining treatment response and local recurrence in nasopharyngeal carcinoma treated curatively. *BMC Cancer.* (2020) 20:193. 10.1186/s12885-020-6664-3 32143592 PMC7060635

[13] Tang LL Huang CL Zhang N Jiang W Wu YS Huang SH Elective upper-neck versus whole-neck irradiation of the uninvolved neck in patients with nasopharyngeal carcinoma: an open-label, non-inferiority, multicentre, randomised phase 3 trial. *Lancet Oncol.* (2022) 23:479–90. 10.1016/S1470-2045(22)00058-4 35240053

[14] Huang L Zhang Y Liu Y Li H Wang S Liang S Prognostic value of retropharyngeal lymph node metastasis laterality in nasopharyngeal carcinoma and a proposed modification to the uicc/ajcc n staging system. *Radiother Oncol.* (2019) 140:90–7. 10.1016/j.radonc.2019.04.024 31195216

[15] Tang L Li L Mao Y Liu L Liang S Chen Y Retropharyngeal lymph node metastasis in nasopharyngeal carcinoma detected by magnetic resonance imaging : prognostic value and staging categories. *Cancer-Am Cancer Soc.* (2008) 113:347–54. 10.1002/cncr.23555 18459174

[16] Guan Y Liu S Wang HY Guo Y Xiao WW Chen CY Long-term outcomes of a phase II randomized controlled trial comparing intensity-modulated radiotherapy with or without weekly cisplatin for the treatment of locally recurrent nasopharyngeal carcinoma. *Chin J Cancer.* (2016) 35:20. 10.1186/s40880-016-0081-7 26879049 PMC4753647

[17] Han F Zhao C Huang SM Lu LX Huang Y Deng XW Long-term outcomes and prognostic factors of re-irradiation for locally recurrent nasopharyngeal carcinoma using intensity-modulated radiotherapy. *Clin Oncol.* (2012) 24:569–76. 10.1016/j.clon.2011.11.010 22209574

[18] Wan Y Tian L Zhang G Xin H Li H Dong A The value of detailed mr imaging report of primary tumor and lymph nodes on prognostic nomograms for nasopharyngeal carcinoma after intensity-modulated radiotherapy. *Radiother Oncol.* (2019) 131:35–44. 10.1016/j.radonc.2018.11.001 30773185

[19] King AD Ahuja AT Leung SF Lam WW Teo P Chan YL Neck node metastases from nasopharyngeal carcinoma: mr imaging of patterns of disease. *Head Neck.* (2000) 22:275–81. 10.1002/(sici)1097-0347(200005)22:33.0.co;2-n 10748451

[20] Ng SH Chang JT Chan SC Ko SF Wang HM Liao CT Nodal metastases of nasopharyngeal carcinoma: patterns of disease on mri and fdg pet. *Eur J Nucl Med Mol Imaging.* (2004) 31:1073–80. 10.1007/s00259-004-1498-9 15007565

[21] King AD Tse GM Ahuja AT Yuen EH Vlantis AC To EW Necrosis in metastatic neck nodes: diagnostic accuracy of ct, mr imaging, and us. *Radiology.* (2004) 230:720–6. 10.1148/radiol.2303030157 14990838

[22] Mao YP Liang SB Liu LZ Chen Y Sun Y Tang LL The n staging system in nasopharyngeal carcinoma with radiation therapy oncology group guidelines for lymph node levels based on magnetic resonance imaging. *Clin Cancer Res.* (2008) 14:7497–503. 10.1158/1078-0432.CCR-08-0271 19010867

[23] Chen M Tang LL Sun Y Mao YP Li WF Guo R Treatment outcomes and feasibility of partial neck irradiation for patients with nasopharyngeal carcinoma with only retropharyngeal lymph node metastasis after intensity-modulated radiotherapy. *Head Neck.* (2014) 36:468–73. 10.1002/hed.23314 23780916

[24] Zhang GY Liu LZ Wei WH Deng YM Li YZ Liu XW. Radiologic criteria of retropharyngeal lymph node metastasis in nasopharyngeal carcinoma treated with radiation therapy. *Radiology.* (2010) 255:605–12. 10.1148/radiol.10090289 20413770

[25] Li YZ Xie CM Wu YP Cui CY Huang ZL Lu CY Nasopharyngeal carcinoma patients with retropharyngeal lymph node metastases: a minimum axial diameter of 6 mm is a more accurate prognostic predictor than 5 mm. *AJR Am J Roentgenol.* (2015) 204:20–3. 10.2214/AJR.14.12936 25539232

[26] Li JJ He LJ Luo GY Liu LZ Huang XX Pan K Fine-needle aspiration of a retropharyngeal lymph node guided by endoscopic ultrasonography. *Endoscopy.* (2015) 47(Suppl. 1):E449–50. 10.1055/s-0034-1392652 26465178

[27] Chan KC Lo YM. Circulating ebv dna as a tumor marker for nasopharyngeal carcinoma. *Semin Cancer Biol.* (2002) 12:489–96. 10.1016/s1044579x02000913 12450734

[28] He Y Yang D Zhou T Xue W Zhang J Li F Epstein-barr virus dna loads in the peripheral blood cells predict the survival of locoregionally-advanced nasopharyngeal carcinoma patients. *Cancer Biol Med.* (2021) 18:888–99. 10.20892/j.issn.2095-3941.2020.0464 33960178 PMC8330545

[29] Chan KC Zhang J Chan AT Lei KI Leung SF Chan LY Molecular characterization of circulating ebv dna in the plasma of nasopharyngeal carcinoma and lymphoma patients. *Cancer Res.* (2003) 63:2028–32. 12727814

[30] Lam W Chan K Lo Y. Plasma epstein-barr virus dna as an archetypal circulating tumour dna marker. *J Pathol.* (2019) 247:641–9. 10.1002/path.5249 30714167 PMC6594142

[31] Ma BB King A Lo YM Yau YY Zee B Hui EP Relationship between pretreatment level of plasma epstein-barr virus DNA, tumor burden, and metabolic activity in advanced nasopharyngeal carcinoma. *Int J Radiat Oncol Biol Phys.* (2006) 66:714–20. 10.1016/j.ijrobp.2006.05.064 17011447

[32] Poh SS Soong YL Sommat K Lim CM Fong KW Tan TW Retreatment in locally recurrent nasopharyngeal carcinoma: current status and perspectives. *Cancer Commun.* (2021) 41:361–70. 10.1002/cac2.12159 33955719 PMC8118589

[33] Lo YM Chan LY Chan AT Leung SF Lo KW Zhang J Quantitative and temporal correlation between circulating cell-free epstein-barr virus DNA and tumor recurrence in nasopharyngeal carcinoma. *Cancer Res.* (1999) 59:5452–5. 10554016

[34] Chan K Woo J King A Zee B Lam W Chan SL Analysis of plasma epstein-barr virus dna to screen for nasopharyngeal cancer. *N Engl J Med.* (2017) 377:513–22. 10.1056/NEJMoa1701717 28792880

[35] Huang CL Sun ZQ Guo R Liu X Mao YP Peng H Plasma epstein-barr virus dna load after induction chemotherapy predicts outcome in locoregionally advanced nasopharyngeal carcinoma. *Int J Radiat Oncol Biol Phys.* (2019) 104:355–61. 10.1016/j.ijrobp.2019.01.007 30682489

[36] Lo YM Chan AT Chan LY Leung SF Lam CW Huang DP Molecular prognostication of nasopharyngeal carcinoma by quantitative analysis of circulating epstein-barr virus dna. *Cancer Res.* (2000) 60:6878–81. 11156384

[37] Liu W Li H Sheng H Liu X Chi P Wang X A randomized controlled trial on evaluation of plasma epstein-barr virus biomarker for early diagnosis in patients with nasopharyngeal carcinoma. *Adv Ther.* (2020) 37:4280–90. 10.1007/s12325-020-01461-4 32780356

[38] Yeh SA Hwang TZ Wang CC Yang CC Lien CF Wang CC Outcomes of patients with nasopharyngeal carcinoma treated with intensity-modulated radiotherapy. *J Radiat Res.* (2021) 62:438–47. 10.1093/jrr/rrab008 33783535 PMC8127674

[39] Qu S Liang ZG Zhu XD. Advances and challenges in intensity-modulated radiotherapy for nasopharyngeal carcinoma. *Asian Pac J Cancer Prev.* (2015) 16:1687–92. 10.7314/apjcp.2015.16.5.1687 25773811

[40] Zhang MX Li J Shen GP Zou X Xu JJ Jiang R Intensity-modulated radiotherapy prolongs the survival of patients with nasopharyngeal carcinoma compared with conventional two-dimensional radiotherapy: a 10-year experience with a large cohort and long follow-up. *Eur J Cancer.* (2015) 51:2587–95. 10.1016/j.ejca.2015.08.006 26318726

[41] Mao YP Tang LL Chen L Sun Y Qi ZY Zhou GQ Prognostic factors and failure patterns in non-metastatic nasopharyngeal carcinoma after intensity-modulated radiotherapy. *Chin J Cancer.* (2016) 35:103. 10.1186/s40880-016-0167-2 28031050 PMC5192583

[42] Hu J Bao C Gao J Guan X Hu W Yang J Salvage treatment using carbon ion radiation in patients with locoregionally recurrent nasopharyngeal carcinoma: initial results. *Cancer-Am Cancer Soc.* (2018) 124:2427–37. 10.1002/cncr.31318 29579324 PMC6001443

[43] Li YQ Tian YM Tan SH Liu MZ Kusumawidjaja G Ong E Prognostic model for stratification of radioresistant nasopharynx carcinoma to curative salvage radiotherapy. *J Clin Oncol.* (2018) 36:891–9. 10.1200/JCO.2017.75.5165 29412781

[44] Lee AW Ng WT Pan JJ Poh SS Ahn YC AlHussain H International guideline for the delineation of the clinical target volumes (ctv) for nasopharyngeal carcinoma. *Radiother Oncol.* (2018) 126:25–36. 10.1016/j.radonc.2017.10.032 29153464

[45] Brouwer CL Steenbakkers RJ Bourhis J Budach W Grau C Gregoire V Ct-based delineation of organs at risk in the head and neck region: dahanca, eortc, gortec, hknpcsg, ncic ctg, ncri, nrg oncology and trog consensus guidelines. *Radiother Oncol.* (2015) 117:83–90. 10.1016/j.radonc.2015.07.041 26277855

[46] Gregoire V Ang K Budach W Grau C Hamoir M Langendijk JA Delineation of the neck node levels for head and neck tumors: a 2013 update. Dahanca, eortc, hknpcsg, ncic ctg, ncri, rtog, trog consensus guidelines. *Radiother Oncol.* (2014) 110:172–81. 10.1016/j.radonc.2013.10.010 24183870

[47] Chen WS Li JJ Hong L Xing ZB Wang F Li CQ. Comparison of mri, ct and 18f-fdg pet/ct in the diagnosis of local and metastatic of nasopharyngeal carcinomas: an updated meta analysis of clinical studies. *Am J Transl Res.* (2016) 8:4532–47. 27904660 PMC5126302

[48] Lam WW Chan YL Leung SF Metreweli C. Retropharyngeal lymphadenopathy in nasopharyngeal carcinoma. *Head Neck.* (1997) 19:176–81. 10.1002/(sici)1097-0347(199705)19:33.0.co;2-# 9142515

[49] Liu N Yan Z Lu Q Wang C. Diagnosis of inguinal lymph node metastases using contrast enhanced high resolution mr lymphangiography. *Acad Radiol.* (2013) 20:218–23. 10.1016/j.acra.2012.09.014 23099240

[50] Chan A Hui EP Ngan R Tung SY Cheng A Ng WT Analysis of plasma epstein-barr virus dna in nasopharyngeal cancer after chemoradiation to identify high-risk patients for adjuvant chemotherapy: a randomized controlled trial. *J Clin Oncol.* (2018). [Epub ahead of print]. 10.1200/JCO.2018.77.7847 29989858

[51] Li H Huang C Chen Q Peng C Zhang R Shen J Lymph-node epstein-barr virus concentration in diagnosing cervical lymph-node metastasis in nasopharyngeal carcinoma. *Eur Arch Otorhinolaryngol.* (2020) 277:2513–20. 10.1007/s00405-020-05937-5 32240363

[52] Smatti MK Al-Sadeq DW Ali NH Pintus G Abou-Saleh H Nasrallah GK. Epstein-barr virus epidemiology, serology, and genetic variability of lmp-1 oncogene among healthy population: an update. *Front Oncol.* (2018) 8:211. 10.3389/fonc.2018.00211 29951372 PMC6008310

[53] Shen Y Zhang S Sun R Wu T Qian J. Understanding the interplay between host immunity and epstein-barr virus in npc patients. *Emerg Microbes Infect.* (2015) 4:e20. 10.1038/emi.2015.20 26038769 PMC4395660

